# Supplementary material for: Epidemiology of alcohol use and alcohol use disorder among female sex workers in Mbeya City, Tanzania
Source: PLOS Glob Public Health. 2024 Apr 25;4(4):e0002794. doi: 10.1371/journal.pgph.0002794 (PMC11045110; doi:10.1371/journal.pgph.0002794)
Supplement: Attachment — Submitted filename: Augustine et al study.docx [file pgph.0002794.s002.docx]

Augustine et al. conducted this cross-sectional study to investigate the prevalence, patterns, and underlying factors associated with alcohol use and AUD among female sex workers in Mbeya, Tanzania. Their study revealed that 86.6% of the female sex workers reported alcohol use in the past 12 months and 85% in the past 30 days. Furthermore, 98.5% of them met AUD criteria. These findings can be useful in policymaking for the country. The study was noteworthy for its use of AUDIT-C and TLFB calendar tools, which were not previously used in valid tools in most of other studies. The results of the pilot study before the main study also add to the credibility of the findings.

Issues:

1. In the introduction, the authors draw attention to the lack of research on Mbeya, Tanzania. Despite this, there exist comprehensive studies such as Beksinska et al.'s.(1) systematic review and meta-analysis, which provide an estimation of the prevalence of harmful alcohol use among female sex workers in low- and middle-income countries, as well as examine associations with various factors. This study, therefore, merits discussion in the subsequent sections of the research.
2. The method employed to select participants in the study is potentially susceptible to selection bias, as the sample was drawn exclusively from individuals identified as being at high risk who were invited to join the registration cohort.
3. Additionally, the study neglected to address the need for long-term follow-up, which could have yielded valuable new insights into this issue (2). Given that previous studies have evaluated the gaps comprehensively, the short study period may not have yielded reliable results
4. The cross-sectional nature of this study limits the ability to establish causality between alcohol use and the factors associated with it.
5. The study relied on self-reported data, which may be subject to recall bias or social desirability bias.
6. The study did not collect data on the types of alcohol consumed, which may be important in understanding the patterns of alcohol use and AUD among female sex workers. Also, they did not collect data on the reasons for alcohol use, which may be important in developing interventions to address harmful alcohol use among female sex workers.

The present study offers valuable insights into the targeted population, which could aid the government in developing more effective prevention plans. However, it is imperative to take into account the limitations and issues that have been identified. These constraints are significant and merit careful consideration. It is my sincere hope that my comments will facilitate the manuscript's revision, leading to an improved final draft. Should you require any further clarification or discussion on any aspect of my feedback, please do not hesitate to contact me.

Yours faithfully,

Farima Safari

1.	Beksinska A, Karlsen O, Gafos M, Beattie TS. Alcohol use and associated risk factors among female sex workers in low- and middle-income countries: A systematic review and meta-analysis. PLOS Glob Public Health. 2023;3(6):e0001216.

2.	Li Q, Li X, Stanton B. Alcohol Use Among Female Sex Workers and Male Clients: An Integrative Review of Global Literature. Alcohol and Alcoholism. 2010;45(2):188-99.
