# Supplementary material for: Epidemiology of alcohol use and alcohol use disorder among female sex workers in Mbeya City, Tanzania
Source: PLOS Glob Public Health. 2024 Apr 25;4(4):e0002794. doi: 10.1371/journal.pgph.0002794 (PMC11045110; doi:10.1371/journal.pgph.0002794)
Supplement: Attachment — Submitted filename: Response to reviewers_5 Mar 24.docx [file pgph.0002794.s003.docx]

**RESPONSE TO REVIEWERS**

**REVIEWER 1 ISSUES:**

1. In the introduction, the authors draw attention to the lack of research on Mbeya, Tanzania. Despite this, there exist comprehensive studies such as Beksinska et al.'s(1). systematic review and meta-analysis, which provide an estimation of the prevalence of harmful alcohol use among female sex workers in low- and middle-income countries, as well as examine associations with various factors. This study, therefore, merits discussion in the subsequent sections of the research.

**Response:**

**Thank you for pointing us to that important reference. We have now included this paper in our discussion.**

2. The method employed to select participants in the study is potentially susceptible to selection bias, as the sample was drawn exclusively from individuals identified as being at high risk who were invited to join the registration cohort.

**Response:**

**Thank you for this comment. Indeed, we agree, the study participants were female sex workers invited to enroll in the cohort registration study. This is a selected group and representative of female sex workers in Mbeya city. Our findings cannot be generalized for general population but certainly could be generalized for Mbeya city female sex workers.**

3. Additionally, the study neglected to address the need for long-term follow-up, which could have yielded valuable new insights into this issue(2). Given that previous studies have evaluated the gaps comprehensively, the short study period may not have yielded reliable results.

**Response:**

**Thanks for the suggestion on the long term follow up, however, in this study, the main aim was to understand the magnitude of alcohol use and alcohol use disorders and characterize the patterns of consumption to inform potential interventions. We suggest that the cross-sectional design would have been ideal for this purpose. One the factors associated with alcohol use and alcohol use disorder it is not possible to determine causality but only associations. This is acknowledged as one of the limitations.**

4. The cross-sectional nature of this study limits the ability to establish causality between alcohol use and the factors associated with it.

**Response:**

**Indeed, thanks for this comment. We agree it is not possible to establish causality but rather associations. We have reported associations and acknowledged the inability to establish causality.**

5. The study relied on self-reported data, which may be subject to recall bias or social desirability bias.

**Response:**

**Indeed, the study relied on self-reported data. We agree there is a risk of underreporting of alcohol use due to social desirability and have acknowledged this. Regarding recall bias we applied the Timeline Follow Back calendar to collect information on recent alcohol consumption and the tools applied relied on the alcohol use in the last 12 months. Alcohol use among female sex workers was extremely high and therefore think the results are still useful albeit the potential limitations.**

6. The study did not collect data on the types of alcohol consumed, which may be important in understanding the patterns of alcohol use and AUD among female sex workers. Also, they did not collect data on the reasons for alcohol use, which may be important in developing interventions to address harmful alcohol use among female sex workers.

**Response:**

**Thanks for this important comment. We agree that types of alcohol consumption and reasons for drinking are important for potential interventions. Previous studies in Tanzania have reported that in urban settings most people consume industrial produced compared to traditional brews. In this study, we did not collect information on traditional brew use, but rather industrial produced brews include the types (beers, wines, spirits). This information was captured in the Timeline Follow Back calendar. We agree reasons for drinking alcohol would have been critical and will acknowledge it as limitation and recommend that potential interventions should factor in the reasons for alcohol use.**

**REVIEWER 2**

Reviewer #2: Dear EIC,
Thank you for your kind invitation,
The manuscript was conducted to evaluate the prevalence, and factors associated with alcohol use among sex workers in Mbeya, Tanzania. the subject was interesting. The studied sample is part of the other study in a cohort, who have a high risk for AIDS. The probability of selection bias is highly important. To estimating the sample size, what is the prevalence of alcohol consumption in the target population? Please specify with related reference.

**Response:**

**Thanks for this observation. Indeed, the study participants were a selected group of female sex workers due to enrol in a cohort study. Female sex workers form one of the hidden populations, so it was ideal, to recruit from the female sex workers mobilized to enrol in the ongoing cohort. The mobilised female sex workers were representative of the population of female sex workers in Mbeya city and as such these findings could be extended to this population. We used the prevalence of alcohol use (14.3%) among female sex workers reported in the study by E. Long et al.,** (3)**to estimate the sample size.**

The most important problem in this study is cross sectional design and high probability of recall bias, especially in these high risk women.

**Response:**

**Indeed, the study relied on self-reported data. We agree there is a risk of underreporting of alcohol use due to social desirability and have acknowledged this. Regarding recall bias we applied the Timeline Follow Back calendar to collect information on recent alcohol consumption and the tools applied relied on the alcohol use in the last 12 months. Alcohol use among female sex workers was extremely high and therefore think the results are still useful albeit the potential limitations. We acknowledge the limitation of the cross sectional nature of the study thus we could not determine the causality.**

**REVIEWER 3**

Reviewer #3:

a) The paragraph of the introduction is long and most sections of the manuscript are too complicated.
   -Some reference numbers are in (), and some in [].

**Response:**

**We have proofread and revised the sentences in the introduction and formatted all the references accordingly.**

b) The order of reference numbers in the text is not observed. for example: For example, reference number 11 is inserted after references 3, 4 and before reference 5.

**Response:**

**We have formatted all the references.**

c) In Sample Size estimation section, the authors should have mentioned the values of p and q and their references.

**Response:**

**The sample size estimation was based on the following assumption, the prevalence of hazardous alcohol use 14.3% as reported from the E. Long et al., study**(4)**.**

d) The authors used abbreviation of female sex workers in *Factors associated with alcohol use* section, while it should have been used earlier in the text.

**Response:**

**Thanks for this observation. We have corrected it.**

e) What were the criteria for classifying people in terms of income?

**Response:**

**Thanks for this comment. In this study, each participant reported individual incomes and, in the analysis, we categorized the income into below and above the median reported income.**

f) The representativeness of the sample is not well defined.

**Response:**

**Thank you for this observation. The study sample is only representative of female sex workers in Mbeya city.**

g) Several analyzes were performed to investigate the relationship, While the mentioned sample size formula belongs to descriptive studies. In addition to this, there are issues related to multiple comparisons.

**Response:**

**Thanks for this comment. The primary aim of the study was to establish the prevalence of risky drinking and patterns of alcohol use among female sex workers in Mbeya city and the secondary aim were to identify factors associated with risky use. There were several outcomes of interest including the risky drinking and those related to patterns of use. We have reported the prevalence of risky drinking and patterns of use and where possible determined the factors associated with risky drinking and abnormal patterns of use such as heavy episodic use. So the sample size enabled us to report the primary aim and the secondary aim where the available data permitted. We assessed multicollinearity and this was non-existent.**

h) Providing a clear definition of Alcohol Use Disorder and other variables is essential.

**Response:**

**Thanks for this comment. We have defined alcohol use disorder on the page six of the manuscript. In this study, we applied AUDIT-C and therefore determined risky drinking. As such Alcohol use disorder in the context of this paper referred to the risky drinking defined by the AUIDIT-C.**

i) The number of references used in the text does not match the number of references at the end of the article.

**Response:**

**We have formatted all the references.**

j) To avoid problems related to multiple comparisons, use appropriate models that allow simultaneous examination of the effect of independent variables on several response variables.

**Response:**

**All multivariable models were assembled by including the apriori decided confounders as described in the data analysis section. And all any other variables were included in the model if they had a p value < 0.20 on bivariate analysis. We assessed multicollinearity and this was non-existent.**

**JOURNAL REQUIREMENTS:**

1. Please include a complete copy of PLOS’ questionnaire on inclusivity in global research in your revised manuscript. Our policy for research in this area aims to improve transparency in the reporting of research performed outside of researchers’ own country or community. The policy applies to researchers who have travelled to a different country to conduct research, research with Indigenous populations or their lands, and research on cultural artefacts. The questionnaire can also be requested at the journal’s discretion for any other submissions, even if these conditions are not met. Please find more information on the policy and a link to download a blank copy of the questionnaire here: https://journals.plos.org/globalpublichealth/s/best-practices-in-research-reporting. Please upload a completed version of your questionnaire as Supporting Information when you resubmit your manuscript.

**Response:**

PLOS` questionnaire on inclusivity in global research has been completed and attached.

2. Tables should not be uploaded as individual files. Please remove these files and include the Tables in your manuscript file as editable, cell-based objects. For more information about how to format tables, see our guidelines:

<https://journals.plos.org/globalpublichealth/s/tables>

**Response:**

Tables have been placed in text after every related paragraph and are editable.

3. We have noticed that you have uploaded Supporting Information files, but you have not included a list of legends. Please add a full list of legends for your Supporting Information files after the references list.

**Response:**

List of legends have been added after reference including supplementary tables and PLOS questionnaire on inclusivity in global research.

REFERENCES

1.	Beksinska A, Karlsen O, Gafos M, Beattie TS. Alcohol use and associated risk factors among female sex workers in low- and middle- income countries : A systematic review and [Internet]. 2023. 1–38 p. Available from: http://dx.doi.org/10.1371/journal.pgph.0001216

2.	Li Q, Li X, Stanton B. Epidemiology Alcohol Use Among Female Sex Workers and Male Clients: An Integrative Review of Global Literature. 2010; Available from: http://www.icap.org/PolicyIssues/

3.	Long JE, Richardson BA, Wanje G, Wilson KS, Shafi J, Mandaliya K, et al. Alcohol use and viral suppression in HIVpositive Kenyan female sex workers on antiretroviral therapy. PLoS One [Internet]. 2020;15(11 November):1–12. Available from: http://dx.doi.org/10.1371/journal.pone.0242817

4.	Long JE, Richardson BA, Wanje G, Wilson KS, Shafi J, Mandaliya K, et al. Alcohol use and viral suppression in HIVpositive Kenyan female sex workers on antiretroviral therapy. PLoS One [Internet]. 2020 Nov 1 [cited 2021 Mar 1];15(11 November). Available from: https://pubmed.ncbi.nlm.nih.gov/33232378/
